# Hepatitis B Virus (HBV) Infection and Re-activation During Nucleos(t)ide Reverse Transcriptase Inhibitor–Sparing Antiretroviral Therapy in a High–HBV Endemicity Setting

**DOI:** 10.1093/ofid/ofy251

**Published:** 2018-10-05

**Authors:** Adam Abdullahi, Olga Mafotsing Fopoussi, Judith Torimiro, Mark Atkins, Charles Kouanfack, Anna Maria Geretti

**Affiliations:** 1 Institute of Infection and Global Health, University of Liverpool, Liverpool, United Kingdom; 2 Chantal Biya International Reference Centre for Research on HIV/AIDS Prevention and Management (CIRCB), Yaoundé, Cameroon; 3 Department of Microbiology, Frimley Park Hospital NHS Foundation Trust, Frimley, United Kingdom; 4 Day Hospital, Yaoundé Central Hospital, Ministry of Public Health, Yaoundé, Cameroon

**Keywords:** hepatitis, lamivudine, mutants, simplification, tenofovir

## Abstract

**Background:**

We monitored the evolution of markers of hepatitis B virus (HBV) infection in virologically suppressed HIV-positive patients switching to nucleoside reverse transcriptase inhibitor (NRTI)–sparing antiretroviral therapy within a randomized trial in Cameroon.

**Methods:**

*** ***HBV surface antigen (HBsAg), HBV DNA, and antibodies against surface (anti-HBs), core (total anti-HBc), and e-antigen (anti-HBe) were measured retrospectively in samples collected at study entry and over 48 weeks after NRTI discontinuation.

**Results:**

Participants (n = 80, 75% females) had a plasma HIV-1 RNA <60 copies/mL, a median CD4 count of 466 cells/mm^3^, and undetectable HBsAg and HBV DNA at study entry. After NRTI discontinuation, 3/20 (15.0%) anti-HBc-negative patients showed evidence indicative or suggestive of incident HBV infection (163 cases/1000 person-years); 6/60 (10.0%) anti-HBc-positive patients showed evidence indicative or suggestive of HBV reactivation (109 cases/1000 person-years). In one case of reactivation, anti-HBs increased from 14 to >1000 IU/L; sequencing showed HBV genotype A3 and 3 escape mutations in surface (Y100C, K122R, Y161FY). Alongside new-onset detection of HBsAg or HBV DNA, 1 patient experienced acute hepatitis and 6 patients experienced mild or marginal increases in serum transaminase levels.

**Conclusions:**

Evolving treatment strategies for sub-Saharan Africa must be accompanied by the formulation and implementation of policy to guide appropriate assessment and management of HBV status.

The World Health Organization (WHO) recommends that HIV-positive adults in sub-Saharan Africa (SSA) be treated with 2 nucleoside reverse transcriptase inhibitors (NRTIs) in combination with a third agent chosen among a non-nucleoside reverse transcriptase inhibitor (NNRTI), the integrase inhibitor dolutegravir, or a boosted protease inhibitor (PI/b) [1]. Recommended NRTI backbones comprise tenofovir disoproxil fumarate (TDF) or zidovudine (ZDV), together with lamivudine (3TC) or emtricitabine (FTC). TDF, 3TC, and FTC also have activity against HBV, and treatment guidelines recommend that HIV/HBV-coinfected patients receive tenofovir for its potent dual antiviral activity and continue this through their initial and subsequent treatment regimens [1, 2]. Outside of SSA, there is established evidence that TDF, FTC, and 3TC reduce the risk of HBV reactivation in patients with a resolved HBV infection who receive immune suppressive treatment [3]. In addition, HIV-positive men who have sex with men (MSM) receiving dually active antiretroviral therapy (ART) in Japan, Western Europe, and North America showed a reduced risk of HBV acquisition in retrospective cohort analyses [4–7].

While triple ART regimens combining 2 NRTIs with a third agent have been the reference model for ART, there is growing interest in exploring alternative regimens that may reduce toxicity and cost, promote compliance, or overcome drug resistance. These include NRTI-sparing regimens that use a PI/b either as monotherapy or in combination with an integrase inhibitor or maraviroc. WHO guidelines list the dual combination of the integrase inhibitor raltegravir with ritonavir-boosted lopinavir (LPV/r) as an alternative second-line regimen (1). Among NRTI- and PI/b-sparing combinations, evidence is emerging for the dual combination of dolutegravir with the NNRTI rilpivirine [8], and there is an interest in combining rilpivirine with the integrase inhibitor cabotegravir as an injectable treatment with prolonged activity [9].

Globally, an estimated 257 million people are chronically infected with HBV, and an estimated 36.7 million people are living with HIV, with substantial overlap [10–12]. SSA bears a disproportionate burden, with 75 million HBV carriers and 25 million HIV-positive people. Whereas HIV programs are established, HBV policies remain underdeveloped across most of the region [13]. Universal childhood vaccination is reducing HBV prevalence in some areas, but the impact remains uneven [14, 15]. In typical programmatic HIV settings, screening for HBsAg is not implemented systematically and management of HIV-positive patients remains commonly blind to HBV status [13, 16–18]. There is also no systematic evaluation of HBV immune status and no systematic adoption of adult vaccination [19].

The aim of the study was to investigate the evolution of markers of HBV infection among patients switching from a programmatic triple ART regimen to a simplified NRTI-sparing regimen within a randomized trial in Cameroon. Stored samples collected at study entry and at regular follow-up visits over 48 weeks were retrieved and tested retrospectively to investigate de novo HBV infection and reactivation.

## METHODS

### Study Population

Participants were HIV-1-positive adults who took part in the Monotherapy in Africa, New Evaluations of Treatment (MANET) trial (NCT02155101). The study was approved by the University of Liverpool Ethics Committee and by the Cameroon National Ethics Committee. Patients eligible to enter MANET were established on 2 NRTIs plus a PI/b for ≥12 months and showed a CD4 count >200 cells/mm^3^ and a plasma HIV-1 RNA load <60 copies/mL on 2 measurements 4 to 12 weeks apart. The initial screening yielded 212 subjects, who next underwent testing to exclude HBsAg positivity. Patients were first tested for HBsAg using Determine (Alere, Kempton Park, South Africa); negative results were confirmed by Architect (Abbott Diagnostics, Maidenhead, United Kingdom). Ten patients tested HBsAg positive by Determine and 1 tested positive by Architect, yielding an HBsAg prevalence in this group of 11/212 (5.2%). A total of 120 patients were randomized (2:1) to (1) switch to monotherapy with once-daily darunavir 800 mg plus ritonavir 100 mg (DRV/r) over 48 weeks (n = 81) or (2) continue triple ART and remain under observation for 24 weeks (n = 39). Patients on monotherapy attended scheduled study visits at weeks 4, 12, 24, 36, and 48, after which they returned to standard of care triple ART with TDF+3TC and LPV/r. Patients who experienced an adverse event graded as either serious or severe [20] and those with virological failure (HIV-1 RNA >400 copies/mL) returned to standard of care triple ART before week 48. Serum biochemistry, full blood counts, CD4 cell counts, and plasma HIV-1 RNA load were measured in the diagnostic laboratory of the Centre Pasteur of Cameroon in Yaoundé. Serum and plasma were separated from whole blood within 2 hours of collection, stored at –80°C, and shipped frozen to the United Kingdom for further HBV testing. A total of 80/81 patients in the monotherapy arm were included in this subanalysis based on the availability of stored samples. At a minimum, all underwent testing for HBsAg, HBV DNA, anti-HBs, and total anti-HBc at study entry and at week 48 or week 36 (or the last available time point before early discontinuation). Subjects with isolated anti-HBc positivity underwent testing for HBV e-antibody (anti-HBe) to provide additional indication of a past infection.

### Laboratory Testing

HBsAg, anti-HBs, total anti-HBc, and anti-HBe were tested at the accredited diagnostic laboratory of Frimley Park Hospital NHS Foundation Trust in the United Kingdom using Architect (Abbott Diagnostics). HBsAg positivity was always confirmed by neutralization, as per recommended diagnostic practice [21, 22]. Anti-HBc positivity was confirmed on the Cobas 8000 analyzer (Roche Diagnostics, Burgess Hill, UK). HBV DNA load was quantified by the M2000sp/RealTime assay (Abbott Molecular, Des Plaines, IL) with a lower limit of quantification of 15 IU/mL. The strength of reactivity of HBV markers was graded to aid interpretation (Supplementary Table 1). Samples with HBV DNA >100 IU/mL underwent sequencing of HBV polymerase and surface as previously described (GenBank access numbers: MH165306, MH165307) [18]. Hepatitis C virus (HCV) RNA was detected with Aptima HCV Quant Dx (Hologic, San Diego, CA).

### Definitions and Analysis

**Figure 1.**
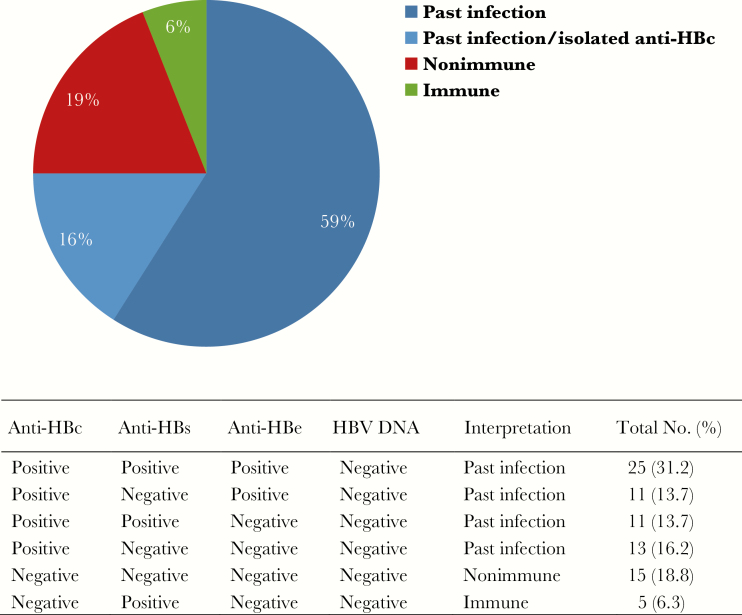
HBV markers among HIV-1-positive subjects who tested hepatitis B surface antigen and HBV DNA negative (n = 80). Abbreviations: anti-HBc, total hepatitis B core antibody; anti-HBe, hepatitis B e antibody; anti-HBs, hepatitis B surface antibody; HBV, hepatitis B virus.

At study entry, all patients tested negative for HBsAg and HBV DNA, and their HBV status was classified as past infection, immune, or nonimmune, as described in Figure 1. At follow-up, (1) incident HBV infection and (2) possible incident HBV infection were defined as detection of HBsAg or HBV DNA (with or without anti-HBc seroconversion) in patients who at study entry either (1) tested negative for all HBV markers or (2) showed anti-HBs as the sole HBV marker [4]. Among subjects who tested anti-HBc positive at study entry, HBV reactivation was defined as either new-onset detection of neutralizable HBsAg reactivity or new-onset detection of HBV DNA in ≥2 separate samples; possible HBV reactivation was defined as new-onset detection of HBV DNA in a single sample. The characteristics of the population at study entry were summarized as either categorical or continuous variables and reported as either proportions or medians with interquartile ranges (IQRs), respectively. Current and nadir CD4 cell counts of anti-HBc-positive patients with or without HBV reactivation were compared by the Wilcoxon-Mann-Whitney test. Incidence and reactivation rates per 1000 person-years with 95% confidence interval (CI) were calculated by dividing the number of patients with the observed event by the total duration of follow-up calculated from each study participant. Analyses were performed with STATA v14 (StataCorp, College Station, TX).

## RESULTS

### Study Population

At study entry, the 80 patients switching to monotherapy had received ART for a median of 7.4 years, including a median of 3.1 years of PI/b-based triple ART (Table 1). Most patients were receiving TDF plus 3TC (70/80, 87.5%), and the predominant PI/b was LPV/r (78/80, 97.5%). The median CD4 count was 466 cells/mm^3^, although previous profound immune suppression was evidenced by a median nadir CD4 cell count of 92 cells/mm^3^. During follow-up, 10/80 (12.5%) patients returned to standard of care triple ART before week 48 due to an adverse event or virological failure.

**Table 1.** Characteristics of the Population at Study Entry, Stratified According to the New-Onset Detection of Markers of HBV Replication Following NRTI Discontinuation

			Markers of HBV Replication	
Characteristic		Total	Yes	No
Total No. (%)		80 (100)	9 (11.3)	71 (88.7)
Females, No. (%)		60 (75.0)	5 (8.3)	55 (91.7)
Age, median (IQR), y		45 (38–52)	48 (36–53)	43 (38–50)
BMI, median (IQR), kg/m^2^		25.5 (21.8–29.1)	24.6 (23.3–29.9)	25.7 (21.1–28.4)
CD4 count, median (IQR), cells/mm^3^		466 (341–615)	535 (328–721)	423 (238–569)
Nadir CD4 count, median (IQR), cells/mm^3^		92 (37–173)	68 (38–229)	96 (36–167)
History of AIDS, No. (%)		11 (13.8)	1 (11.1)	10 (14.1)
Haemoglobin, median (IQR), g/dL		12.3 (11.5–13.2)	12.6 (12.5–13.8)	12.3 (11.4–13.1)
Platelets, median (IQR), cells ×10^3^/mm^3^		209 (173–256)	216 (189–244)	230 (191–275)
Bilirubin, median (IQR), mg/L		5.3 (3.8–7.7)	6.0 (4.1–7.7)	6.6 (4.1 9.0)
Alkaline phosphatase, median (IQR), U/L		95 (77–123)	144 (77–177)	116 (92–138)
AST, median (IQR),^a^ U/L		23 (18–30)	27.0 (19–36)	29 (24–35)
ALT, median (IQR),^a^ U/L		20 (15–28)	22.0 (16–32)	24 (18–33)
NRTI backbone, No. (%)	TDF + 3TC	70 (87.5)	7 (10.0)	63 (90.0)
	ZDV + 3TC	4 (5.0)	0 (0)	4 (100)
	ABC + TDF	1 (1.3)	0 (0)	1 (100)
	ABC + ddI	5 (6.2)	2 (40.0)	3 (60.0)
ART duration, median (IQR), y		7.4 (5.3–9.5)	9.1 (7.1–11.0)	7.3 (5.3–9.8)
PI/r duration, median (IQR), y		3.1 (1.3–5.5)	4.0 (2.2–5.2)	2.6 (1.3–5.8)
3TC duration, median (IQR), y		6.8 (5.0–9.3)	8.8 (6.8–9.3)	6.3 (4.5–9.1)
TDF duration, median (IQR), y		2.9 (1.5–4.6)	3.6 (1.6–4.6)	2.8 (1.2–4.6)

Abbreviations: 3TC, lamivudine; ABC, abacavir; ALT, alanine transaminase; ART, antiretroviral therapy; AST, aspartate transaminase; BMI, body mass index; ddI, didanosine; HBV, hepatitis B virus; NRTI, nucleos(t)ide reverse transcriptase inhibitors; PI/r, ritonavir-boosted protease inhibitor; TDF, tenofovir disoproxil fumarate; ZDV, zidovudine.

^a^The laboratory reference ranges for AST and ALT were 10–40 IU/L and 8–50 IU/L, respectively.

### HBV Status at Study Entry

All patients were HBsAg and HBV DNA negative at study entry. Overall, 60/80 (75.0%) had evidence of a past HBV infection based on total anti-HBc (Figure 1). Of these, 47/60 (78.3%) also had anti-HBs and/or anti-HBe, whereas 13/60 (21.7%) had isolated anti-HBc. Anti-HBs were detected as the sole HBV marker in 5/80 (6.3%) patients, with median levels (range) of 39 (12–179) IU/L. A further 15/80 (18.8%) patients tested negative for all HBV markers.

### Evolution of HBV Status During Follow-up

#### Incident and Possible Incident HBV Infection

Among anti-HBc-negative patients, 3/20 (15.0%) showed profiles indicative or suggestive of incident HBV infection, totaling 163 cases per 1000 person-years (95% CI, 139–190). Among the 15 patients lacking all HBV makers at study entry, 1 (6.7%) experienced de novo HBV infection. The patient (CUI-125) (Table 2) experienced a flu-like illness at week 23 followed by icterus and grade 4 ALT and AST elevations at week 24, with raised total bilirubin (132 mg/L) and alkaline phosphatase (309 IU/L) and preserved liver synthetic function. The patient returned to standard of care triple ART at week 26, made a full recovery by week 32, and was recalled at weeks 36 and 105 for poststudy assessment. Retrospectively, the patient was negative for all HBV markers at study entry. Strong HBsAg reactivity and HBV DNA levels of 28 259 IU/mL and 232 569 IU/mL were detected at weeks 12 and 24, respectively. HBV DNA sequencing showed genotype E, with no major mutations in polymerase or surface. HBsAg and HBV DNA became undetectable by week 26, and anti-HBc seroconversion was demonstrated at week 105, in the absence of detectable anti-HBs and anti-HBe.

**Table 2.** HBV Markers Among Subjects With Incident (Patient CUI-125) or Possible Incident (Patients CUI-156 and CUI-218) HBV Infection^a^

Marker	CUI-125 (Female, Aged 29)^b^ Week							CUI-156 (Male, Aged 54) Week						CUI-218 (Female, Aged 65) Week					
	0	4	12	24	26	36	105	0	4	12	24	36	48	0	4	12	24	36	48
HBsAg	-	-	+++	++++	-	-	-	-			-	++	+	-		-	-	-	
Anti-HBs	-						-	+			++		+	+		+		+++	
Anti-HBc	-		-				+	-	-	-	+		+	-		-	-	++	
Anti-HBe	-		-	-			-	-			-		-	-		-		+	
HBV DNA	-	-	++++	+++++			-	-	-	-	-		-	-		-	+	+	
AST U/L	19	23	19	661				27	30	41	42	37	31	36	21	21	23	19	102
ALT U/L	28	12	20	824				27	21	35	59	39	44	20	16	18	19	16	109
HIV-1 RNA^c^	UD		UD	UD				UD		UD	UD	UD	UD	UD		UD	105	UD	
CD4 count^d^	340			426				338			268		337	535			667		674
NRTIs	TDF 3TC	None	None	None	TDF 3TC	TDF 3TC	TDF 3TC	TDF 3TC	None	None	None	None	None	TDF 3TC	None	None	None	None	None

Abbreviations: ALT, alanine transaminases; anti-HBc, total hepatitis B core antibody; anti-HBe, hepatitis B e antibody; anti-HBs, hepatitis B surface antibody; AST, aspartate transaminase; 3TC, lamivudine; HBsAg, hepatitis B surface antigen; HBV, hepatitis B virus; NRTIs, nucleos(t)ide reverse transcriptase inhibitors; TDF, tenofovir disoproxil fumarate; UD, undetectable (<60 copies/mL).

^a^After study entry, study visits were planned at weeks 4, 12, 24, 36, and 48. Given the retrospective nature of the analysis, stored samples for HBV testing were not available from all study visits; missing time points are blank.

^b^Patient returned to standard of care triple ART at week 26 due to acute hepatitis and was recalled at weeks 36 and 105 for poststudy assessment.

^c^Plasma HIV-1 RNA load in copies/mL.

^d^CD4 count in cells/mm^3^.

Among the 5 subjects showing only anti-HBs at study entry, 2 (40.0%) with anti-HBs levels of 17 IU/L and 12 IU/L, respectively, showed profiles suggestive of incident HBV infection (Table 2). In patient CUI-156, anti-HBc seroconversion was first detected at week 24, followed by HBsAg detection at weeks 36 and 48. ALT levels increased by up to 2-fold relative to study entry, without other laboratory abnormalities or clinical adverse events. HBV DNA was not detected at multiple sampling points (weeks 4, 12, 24, and 48) (Table 2). In patient CUI-218, HBV DNA was detected first at week 24 (85 IU/mL) and then at week 36 (20 IU/mL). Anti-HBc and anti-HBe seroconversion was observed at week 36, with an increase in anti-HBs levels to 197 IU/L. No HBsAg was detected at multiple sampling points (weeks 12, 24, and 36) (Table 2). ALT and AST levels showed a grade 1 elevation at week 48, with the ALT increasing by >5-fold relative to study entry, without other laboratory abnormalities or clinical adverse events. HBV markers were not measured at this time due to the unavailability of stored samples. HCV RNA was not detected in this group.

#### HBV Reactivation and Possible HBV Reactivation

Among anti-HBc-positive patients, 6/60 (10.0%) showed profiles indicative or suggestive of HBV reactivation, totaling 109 cases per 1000 person-years (95% CI, 90–131). Median CD4 counts in anti-HBc-positive subjects with and without HBV reactivation (IQR) were 508 (274–637) vs 420 (330–568) cells/mm^3^, respectively (*P* = .79); in the same population, median nadir CD4 counts (IQR) were 59 (29–108) vs 92 (37–167) cells/mm^3^, respectively (*P* = .31). Patients CUI-030, CUI-143, CUI-238, and CUI-321 had anti-HBs levels ranging between 14 and 191 IU/L at study entry, whereas patients CUI-052 and CUI-213 had no detectable anti-HBs. Profiles indicative of HBV reactivation were observed in 3 subjects (CUI-030, CUI-052, and CUI-143) (Table 3). Patient CUI-030 showed detectable HBV DNA at weeks 12 (qualitative detection < 15 IU/mL), 24 (49 IU/mL), and 48 (83 IU/mL); HBsAg was only detected at week 48, accompanied by increased anti-HBc reactivity. Anti-HBs were 191 IU/L at study entry and 179 IU/L at week 36. Transaminase levels were not increased at any study visit. The patient reported moderate arthralgia at an unscheduled visit at week 32; the malaria test was negative. Patient CUI-052 only had 2 sampling points available for testing of HBV markers, at weeks 24 and 36. HBV DNA was detected at weeks 24 (84 IU/mL) and 36 (247 IU/mL). HBsAg was not detected at week 36, although ALT levels showed a marginal increase (~2-fold relative to study entry), without other laboratory abnormalities or clinical adverse events. In patient CUI-143, HBsAg was detected at week 36, following negative tests at weeks 12 and 24. No HBV DNA was detected at multiple sampling points (weeks 4, 12, 24, and 36). Anti-HBs levels were 169 IU/L at study entry and 211 IU/L at week 12, declining to 69 IU/L at week 36. Transaminase levels showed a grade 1 elevation at week 12, without other laboratory abnormalities or clinical adverse events; the ALT raised by <2-fold relative to study entry.

**Table 3.** HBV Markers Among Subjects With HBV Reactivation^a^

Markers	CUI-030 (Female, Aged 31) Week						CUI-052 (Male, Aged 41) Week						CUI-143 (Female, Aged 51) Week					
	0	4	12	24	36	48	0	4	12	24	36	48	0	4	12	24	36	48
HBsAg	-			-	-	+	-				-		-		-	-	+	
Anti-HBs	+++			++	+++		-						+++		+++		++	
Anti-HBc	+					++	+			+			+					
Anti-HBe^b^	-			+	-		-						-			-	-	
HBV DNA	-		+	++		++	-			+	++		-	-	-	-	-	
AST U/L	18	24	12	15	15	14	35	35	26	24	46	30	67	62	92	40	55	26
ALT U/L	22	16	17	11	12	13	25	25	25	28	49	30	61	78	102	38	65	26
HIV-1 RNA^c^	UD		UD	UD	UD	UD	UD		UD	UD	UD	3715	UD		UD	UD	UD	
CD4 count^d^	317			360		409	734			660			604			657		600
NRTIs	TDF 3TC	None	None	None	None	None	TDF 3TC	None	None	None	None	None	TDF 3TC	None	None	None	None	None

Abbreviations: ALT, alanine transaminases; anti-HBc, total hepatitis B core antibody; anti-HBe, hepatitis B e antibody; anti-HBs, hepatitis B surface antibody; AST, aspartate transaminase; 3TC, lamivudine; HBsAg, hepatitis B surface antigen; HBV, hepatitis B virus; NRTIs, nucleos(t)ide reverse transcriptase inhibitors; TDF, tenofovir disoproxil fumarate; UD, undetectable (<60 copies/mL).

^a^After study entry, study visits were planned at weeks 4, 12, 24, 36, and 48. Given the retrospective nature of the analysis, stored samples for HBV testing were not available from all study visits; missing time points are blank.

^b^Weak anti-HBe reactivity was transiently detected at week 24 and was confirmed by repeat testing of the same sample.

^c^Plasma HIV-1 RNA load in copies/mL.

^d^CD4 count in cells/mm^3^.

A possible HBV reactivation was observed in 3 patients (CUI-213, CUI-238, and CUI-321) showing detection of HBV DNA at a single time point, in the absence of HBsAg (Table 4). HBV DNA levels ranged between 20 and 60 IU/mL in this group. Transaminase levels remained within the laboratory reference range, although ALT levels increased by around 2-fold relative to study entry, without other laboratory abnormalities or clinical adverse events. In patient CUI-238, anti-HBs levels were 14 IU/L at study entry and 49 IU/L at week 36 when HBV DNA was detected. Patient CUI-321 showed detectable HBV DNA at week 48, coinciding with a marked increase in anti-HBs levels from 21 IU/L at study entry to >1000 IU/L at week 48. In this patient, sequence data from week 48 showed genotype A3 with no resistance mutations in polymerase; the major hydrophobic region (MHR) of surface showed Y100C and Y161FY; in addition, arginine (R) was present at position 122 instead of lysine (K), as per the consensus sequence of genotype A3. HCV RNA was not detected in this group.

**Table 4.** HBV Markers Among Subjects With Possible HBV Reactivation^a^

Markers	CUI-213 (Female, Aged 51)^b^ Week						CUI-238 (Female, Aged 47) Week						CUI-321 (Male, Aged 48) Week					
	0	4	12	24	36	48	0	4	12	24	36	48	0	4	12	24	36	48
HBsAg	-		-				-				-		-					-
Anti-HBs	-		-				+				+		+				+	++++
Anti-HBc	+						++				++		+				+	
Anti-HBe	+						+						-					
HBV DNA	-	-	+				-		-		+		-		-		-	+
AST U/L	19	12	10	23	15	15	33	24	26	31	28	24	14	12	14	16	19	21
ALT U/L	15	13	17	16	14	30	17	14	35	33	10	26	8	11	11	23	17	21
HIV-1 RNA^c^	UD		UD	UD	UD	UD	UD		UD	UD	496	1098	UD		UD	UD	UD	UD
CD4 count^d^	566			728		680	449			495		484	143			154		192
NRTIs	TDF 3TC	None	None	TDF 3TC	TDF 3TC	TDF 3TC	TDF 3TC	None	None	None	None	None	TDF 3TC	None	None	None	None	None

Abbreviations: ALT, alanine transaminases; anti-HBc, total hepatitis B core antibody; anti-HBe, hepatitis B e antibody; anti-HBs, hepatitis B surface antibody; AST, aspartate transaminase; 3TC, lamivudine; HBsAg, hepatitis B surface antigen; HBV, hepatitis B virus; NRTIs, nucleos(t)ide reverse transcriptase inhibitors; TDF, tenofovir disoproxil fumarate; UD, undetectable (<60 copies/mL).

^a^After study entry, study visits were planned at weeks 4, 12, 24, 36, and 48. Given the retrospective nature of the analysis, stored samples for HBV testing were not available from all study visits; missing time points are blank.

^b^Patient returned to standard of care triple ART at week 19 due to an adverse event and was recalled at weeks 24, 36, and 48 for poststudy assessment.

^c^Plasma HIV-1 RNA load in copies/mL.

^d^CD4 count in cells/mm^3^.

## DISCUSSION

This study was the first to assess the evolution of markers of HBV infection in HIV-1-positive adults who introduced NRTI-sparing ART in SSA. Patients were at risk of both clinically manifest acute hepatitis B and more subtle evidence of resumed HBV replication, a finding that bears implications for researchers and policy makers alike. Investigating or rolling out in SSA regimens that omit HBV-active NRTIs requires adoption of measures and interventions to address the high rates of HBV exposure, including (1) systematic HBV screening including but not limited to HBsAg, (2) vaccination of nonimmune subjects, and (3) monitoring of subjects with a previous HBV infection for evidence of reactivation. Across most of SSA, such measures and interventions currently have limited implementation in routine practice [10, 13–16, 19].

The WHO recommends that HIV-1-positive patients in SSA receive ART regimens containing either TDF plus 3TC or FTC, or ZDV plus 3TC if HBsAg negative [1, 2]. 3TC alone is expected to exert at least partial prophylactic activity against HBV acquisition [5] and reactivation [23]. Our data, combined with those from the published literature, indicate that NRTI-sparing and other regimens omitting both tenofovir and 3TC or FTC would carry a substantial risk of HBV infection and reactivation. HBsAg prevalence is 8.8% across SSA and is highest in Central and West Africa [24]. HBV infection rates are similarly high among HIV-positive people in the region [12]. In Cameroon, although data are heterogeneous, HBsAg prevalence is around 10% in the general population [25] and among HIV-positive patients [17]. HBV transmission occurs early in life across SSA, commonly through horizontal spread among young children [26]. Consistent with this assumption, most HBsAg-negative patients in our study had evidence of a previous HBV infection and were therefore at risk of reactivation.

In high-income countries, HBV acquisition in the setting of HIV infection has been investigated predominantly among MSM. In Amsterdam, the overall incidence rate was 11/1000 person-years in this group, ranging from 29/1000 person-years in the absence of HBV-active drugs to 14/1000 when only 3TC was used, and 1.4/1000 in the presence of tenofovir [5]. A similar protective effect of dually active ART was reported among MSM in Japan [4] and North America [6] and among HIV-1-positive patients in Switzerland [7]. Previous case reports have also reported HBV reactivation in the context of HIV-induced immune suppression [27–29]. CD4 cell counts were overall satisfactory in our cohort, without a significant difference between patients with and without HBV reactivation. Patients, however, had experienced low CD4 cell counts before immune reconstitution on ART, and there was a trend for lower nadir CD4 counts in patients with HBV reactivation relative to those without. The likelihood of HBV reactivation during immune suppression is effectively reduced by the use of HBV-active antivirals [3]. Dually active ART has also been shown to reduce HBV DNA detection among HBsAg-negative/anti-HBc-positive patients with HIV infection [30]. Clinical trials that have evaluated NRTI-sparing ART strategies in high-income countries, including previous trials of PI/b monotherapy, have not reported on the risk of HBV infection or reactivation in their participants [31–33].

HBV reactivation carries a risk of liver disease ranging from mild to fatal [34]; the risk of liver disease progression is augmented by HIV coinfection [35]. Anti-HBs reduces the risk of HBV reactivation in populations with previous infection [30, 36]; yet, 2 subjects in our study showed persuasive evidence of HBV reactivation despite anti-HBs levels >100 IU/L. Mutations in the MHR region of HBsAg can allow escape from both antibody-mediated neutralization (including vaccine escape) and HBsAg detection in diagnostic assays [29, 35, 37]. In 1 patient, anti-HBs levels increased >1000 IU/L, coinciding with the detection of HBV DNA, in the absence of HBsAg. The viral sequence showed the major MHR mutations Y100C and Y161FY; in addition, the patient harbored HBV genotype A3, which circulates in Cameroon and carries arginine (R) rather than lysine (K) at surface position 122. Y100C and K122R have been previously recognized in the context of HBsAg-negative HBV infection (“occult hepatitis B”) [38, 39]; Y100C has also been previously described in a patient with co-circulating HBsAg and anti-HBs [40]. When occurring in isolation, neither mutation appears to confer significant escape potential [41, 42]; a more substantial effect has been proposed for multiple mutations occurring in combination [42]. The impact of HBV genetic evolution and escape in the context of HBV hyperendemicity warrants further investigations in larger numbers of patients.

Universal vaccination programs have variable coverage across SSA [43]. Cameroon introduced infant vaccination in 2005, and coverage with 3 vaccine doses was 85% in 2016 [43]. In our study, about 1 in 5 patients lacked evidence of HBV immunity. This should be interpreted in light of the lack of an adult catch-up vaccination program, as also indicated by the poor vaccination coverage of health care workers [44]. A further 5 patients had anti-HBs as the only detectable HBV marker at study entry, which may be taken to indicate previous vaccination; their age (38–65 years) made them unlikely recipients of infant vaccination, and the subjects did not report vaccination, although we have previously noted that patients’ recall of vaccination history is generally poor [45]. It is noteworthy that 2 of the 5 subjects, both with low anti-HBs levels, showed evidence of possible incident HBV infection during follow-up. This may indicate that the antibodies were insufficiently protective. An alternative hypothesis is that anti-HBs may have reflected a previous HBV exposure, despite absence of anti-HBc. Although antibodies against HBV core usually appear shortly after infection and remain positive lifelong, anti-HBc negativity has been reported despite evidence of HBV replication, typically in the context of immune suppression, and including cases with detectable anti-HBs [46–48]. Thus, it cannot be excluded that the 2 patients with possible incident HBV infection might in fact have experienced a reactivation, although it should be noted that anti-HBc did appear during follow-up alongside detection of HBsAg or HBV DNA.

This study has limitations. It was advantageous to have access to samples collected within a clinical trial, which provided good retention into follow-up, laboratory test results, and well-preserved samples. However, the retrospective nature of the study restricted sample availability. HBV DNA sequences were recovered in 2 subjects; due limited sample volumes, we were unable to increase testing sensitivity at HBV DNA levels <100 IU/mL. A further consideration is that poststudy samples could only be collected from 1 patient. We also lacked a control group continuing triple ART, and further studies should aim to quantify the protective effect of dually active ART against HBV infection and reactivation in SSA. Finally, standard serological measures were used to categorize patients with incident HBV infection or reactivation, and this must be regarded as a simplified approach when considering the complexity of the HBV marker profiles we observed, the multiple possible phenotypes of HBV infection, and the modulatory effect of HIV coinfection.

In summary, this is the first study to report on the risk of HBV infection and HBV reactivation among HIV-1-positive patients who discontinue HBV-active agents in SSA. Results clearly indicate that HIV-1 treatment strategies for the region must take into consideration available infrastructure for assessing, monitoring, and appropriately managing HBV status and must consider level of access to adult HBV immunization.

## Supplementary Data

Supplementary materials are available at *Open Forum Infectious Diseases* online. Consisting of data provided by the authors to benefit the reader, the posted materials are not copyedited and are the sole responsibility of the authors, so questions or comments should be addressed to the corresponding author.

ofy251_suppl_supplementary_table_1

## Notes

***Prior presentation*.** Findings were presented at the Liver Meeting 2017 (Washington DC); abstract #1919.
