# Corrigendum: Hepatitis B Virus (HBV) Infection and Re-activation During Nucleos(t)ide Reverse Transcriptase Inhibitor–Sparing Antiretroviral Therapy in a High–HBV Endemicity Setting

**DOI:** 10.1093/ofid/ofz142

**Published:** 2019-04-03

**Authors:** Adam Abdullahi, Olga Mafotsing Fopoussi, Judith Torimiro, Mark Atkins, Charles Kouanfack, Anna Maria Geretti

The sentence: “Patients eligible to enter MANET were established on 2 NRTIs plus a PI/b for ≥12 months and showed a CD4 count >200 cells/mm3 and a plasma HIV-1 RNA load.” should read: “Patients eligible to enter MANET were established on 2 NRTIs plus a PI/b for ≥12 weeks and showed a CD4 count >100 cells/mm3 and a plasma HIV-1 RNA load.” This has been updated in the original article.

