# Supplementary material for: Hepatitis B Virus (HBV) Infection and Re-activation During Nucleos(t)ide Reverse Transcriptase Inhibitor–Sparing Antiretroviral Therapy in a High–HBV Endemicity Setting
Source: Open Forum Infect Dis. 2018 Oct 5;5(10):ofy251. doi: 10.1093/ofid/ofy251 (PMC6201150; doi:10.1093/ofid/ofy251)
Supplement: ofy251_suppl_supplementary_table_1 [file ofy251_suppl_supplementary_table_1.docx]

**Supplementary Table 1.** Reactivity keys for HBV markers

| HBsAg | |
| --- | --- |
| s/c | key |
| <1 | - |
| 1-10 | + |
| 10-100 | ++ |
| >100 | +++ |
| >1000 | ++++ |
| anti-HBc | |
| s/c | key |
| <1 | - |
| 1-5 | + |
| 5-10 | ++ |
| anti-HBs | |
| IU/L | key |
| <10 | - |
| 10-50 | + |
| 50-100 | ++ |
| >100 | +++ |
| anti-HBe | |
| s/c | key |
| >1 | - |
| <1 | + |
| HBV DNA | |
| IU/mL | key |
| <10 | - |
| 10-100 | + |
| 100-1000 | ++ |
| 1000-10000 | +++ |
| 10000-100000 | ++++ |
| >100000 | +++++ |

s/c= signal to cut-off ratio
